# Evaluation of ^18^F-FDG uptake for detecting lymph node metastasis of gastric cancer: a prospective pilot study for one-to-one comparison of radiation dose and pathological findings

**DOI:** 10.1186/s12957-015-0743-y

**Published:** 2015-12-02

**Authors:** Yasuhiro Okumura, Susumu Aikou, Haruna Onoyama, Keiichi Jinbo, Yukinori Yamagata, Kazuhiko Mori, Hiroharu Yamashita, Sachiyo Nomura, Miwako Takahashi, Keitaro Koyama, Toshimitsu Momose, Hiroyuki Abe, Keisuke Matsusaka, Tetsuo Ushiku, Masashi Fukayama, Yasuyuki Seto

**Affiliations:** Department of Gastrointestinal Surgery, The University of Tokyo, 7-3-1 Hongo, Bunkyo-ku, Tokyo 113-8655 Japan; Department of Nuclear Medicine, The University of Tokyo, 7-3-1 Hongo, Bunkyo-ku, Tokyo 113-8655 Japan; Department of Pathology, The University of Tokyo, 7-3-1 Hongo, Bunkyo-ku, Tokyo 113-8655 Japan

**Keywords:** ^18^F-FDG, Gastric cancer, Lymph node metastasis, PET/CT, Navigation surgery

## Abstract

**Background:**

Gastric cancer exhibits various degrees of fluorine F-18 fluorodeoxyglucose (^18^F-FDG) uptake on positron emission tomography/computed tomography (PET/CT). We evaluated the relationship between ^18^F-FDG uptake and the presence/absence of metastasis in individual lymph nodes (LN) on a one-to-one basis.

**Methods:**

We analyzed 21 patients with gastric cancer. We injected ^18^F-FDG intravenously in the morning, and gastrectomy with LN dissection was performed in the afternoon of the same day. Radiation doses were measured at each LN using a well-type counter, and we then compared ^18^F-FDG uptake, the shortest diameter, and pathological examination results for each LN.

**Results:**

In our study, 906 LNs were analyzed, including 115 metastatic LNs. Metastatic LNs showed significantly higher ^18^F-FDG uptake (*P* < 0.0001), and were significantly enlarged (*P* < 0.0001). The receiver operating characteristics (ROC) curve had a larger area under the curve (0.71) for ^18^F-FDG uptake than for the shortest LN diameter (0.60). Considering histology, the ROC curve for intestinal type adenocarcinoma had a larger area under the curve than that for diffuse type (0.75 vs 0.61).

**Conclusions:**

F-FDG uptake is potentially a more useful variable than LN diameter for discriminating between LN with and without metastasis, especially in intestinal type gastric cancer cases.

## Background

In gastric cancer, lymph node (LN) metastasis is an important prognostic factor [1, 2]. Among patients with R0 resection for gastric cancer, LN status was the most important independent prognostic factor, followed by the pT category, surgical complication, and distant metastasis [1]. Therefore, accurate knowledge of LN status would be helpful for predicting prognosis and planning the extent of lymphadenectomy. Enhanced computed tomography (CT), which is routinely performed to evaluate LN metastasis in gastric cancer, has a sensitivity of 80.0 % and a specificity of 77.8 %, based on the size of LN [3]. However, Monig et al. reported LN size to be an unreliable indicator of LN metastasis in patients with gastric cancer [4]. As compared to CT scans, fluorine F-18 fluorodeoxyglucose positron emission tomography and computed tomography (^18^F-FDG PET/CT) shows lower sensitivity and higher specificity for evaluating regional LN metastasis [5]. However, low sensitivity may result from low spatial resolution of both PET scanning and PET/CT scan [3, 5–7]. Considering the difficulty to diagnose LN metastasis preoperatively, prophylactic LN dissection is regarded as essential to curative resection for gastric cancer, resulting in the dissection of non-metastatic LNs.

^18^F-FDG has been used for not only preoperative diagnosis but also intraoperative diagnosis and navigation surgery using intraoperative gamma probe [8–10]. This navigation system during gastric cancer surgery can be planned, if the radiation dose of ^18^F-FDG shows nodal involvement precisely. To our knowledge, there are no reports comparing pathological findings and ^18^F-FDG uptake on a one-to-one basis.

The aim of this study was to clarify the diagnostic power of ^18^F-FDG by investigating the one-to-one relationship between the ^18^F-FDG uptake of each dissected LN and the corresponding pathological results.

## Methods

### Patients

Study patients were recruited between July 2012 and September 2013 at the Department of Gastrointestinal Surgery, the University of Tokyo Hospital, Japan, for a prospective pilot study. Criteria for inclusion in this study were (1) adenocarcinoma of the stomach confirmed by pathological examination, (2) diagnosis of advanced gastric cancer based on preoperative CT scan or endoscopic examination results, (3) necessity of gastrectomy for curative or palliative intent, (4) age 85 years or younger, (5) normal renal function, and (6) European clinical oncology group performance status (ECOG-PS) ≦1. Exclusion criteria were (1) diabetes mellitus, (2) any severe ongoing comorbidity, (3) prior malignant diseases, and (4) synchronous malignancies other than gastric cancer. For patients who meet these criteria, we injected ^18^F-FDG on the day of surgery, took ^18^F-FDG PET/CT in the morning, and measured radiation dose of each LN after harvesting LNs by surgery in the afternoon. We only included patients with advanced gastric cancer since ^18^F-FDG PET/CT suffers from low detection rate of LN involvement for early gastric cancer [11].

### Ethics statement

The scientific protocol was approved by the local ethics committee (Graduate School of Medicine and Faculty of Medicine, the University of Tokyo, no. 3799). Written informed consent for participating this study and publishing was obtained from all participants. This trial was registered in the UMIN Clinical Trial Registry (UMIN 000013934, http://www.umin.ac.jp/ctr/).

### FDG-PET/CT study

In the morning of the day of gastrectomy, ^18^F-FDG was injected intravenously 3–4 h prior to surgery, and PET/CT scans were obtained. Patients fasted for at least 5 h before undergoing FDG-PET, and a blood sugar level under 150 mg/dL was required. Each patient received 296 MBq of intravenous FDG. Imaging was then performed 50 min later using an Aquiduo PET/CT scanner (Toshiba Medical Systems, Otawara, Japan). This scanner contains 24,336 lutetium oxyorthosilicate (LSO) crystals in 39 detector rings and has an axial field of view of 16.2 cm and 82 transverse slices with a 2.0 mm thickness. The intrinsic full width at half-maximum (FWHM) spatial resolution in the center of the field of view is ~4.3 mm, and the FWHM axial resolution is 4.7 mm. The sinogram was acquired in the three-dimensional mode. The CT scan was performed with a tube current of 50 mA and a tube voltage of 120 kV for attenuation correction, and one 2.5-min emission scan per position was acquired. Images were reconstructed using Fourier rebinning ordered subset expectation maximization iterative reconstruction, with two iterations and eight subsets, and a 4-mm FWHM Gaussian filter was applied. The data were collected in a 128 × 128 × 41 matrix with a voxel size of 2.0 × 2.0 × 4.0 mm.

PET/CT images were visually evaluated by two of the authors, both of whom are experienced nuclear medicine physicians (MT and KK). The maximum activity concentration within the lesions of interest was determined and expressed as the maximal standardized uptake value (SUV max). All SUV measurements were normalized for patient body weight and for the time elapsed from injection until data acquisition. If the PET/CT scan showed distant metastasis, we reviewed the indications for the scheduled gastrectomy. We determined the sensitivity, specificity, positive predictive value (PPV), and negative predictive value (NPV) of ^18^F-FDG PET as described in a previous report [7]. We classified regional LNs into three groups: LNs along the lesser curvature, LNs along the greater curvature, and other regional LNs (LNs in suprapancreatic area and hepatoduodenal ligament). LNs were considered positive or negative on the basis of the group as a whole.

### Radiation dose measurements for individual LNs

After ^18^F-FDG PET/CT scan in the morning, gastrectomy with LN dissection was performed in the afternoon. All LNs were harvested from the surgical specimen before formalin fixation. We measured the radiation dose of each LN using a CAPRAC-t well-type counter (Capintec, Inc., Pittsburgh, PA, USA) with the energy window set at 464.7–557.3 keV before formalin fixation and staining. The energy window was determined by the FWHM peak of the 511 keV radiation spectrum, in order to precisely measure the radiation dose of ^18^F-FDG. Since it took 40–50 s to prepare for the radiation dose measurement in each LN, we set the count time at 30 s to assure that the time between the first LN and the last LN measurement would be within the half-life of ^18^F-FDG (109.8 min). In addition, we determined the weight and shortest diameter of each LN before fixation. To assess ^18^F-FDG uptake of each LN by well-type counter, the modified standardized uptake value of each LN (modified SUV) was calculated using the following formula: modified SUV = CCF × *C*_dc_/(*d*_i_/*w*), where CCF is the cross-calibration factor, *C*_dc_ is the decay-corrected tracer tissue concentration normalized for the time elapsed from ^18^F-FDG injection until data acquisition (in counts per second per gram), *d*_i_ is the injected dose (in becquerels), and *w* is the patient’s body weight (in grams). CCF is the ratio of the radioactivity (count per second) measured with the well-type counter to those (in becquerels) obtained with the dose calibrator. In this study, we determined CCF to be 5.8 based on our measurements using test tubes filled with ^18^F-FDG solution, the dose calibrator, and the well-type counter. We also compared the diagnostic usefulness of LN size and ^18^F-FDG uptake.

Surgical specimens including the excised stomach and LNs were examined by an experienced pathologists (KM, HA, and TU) with no knowledge of either the ^18^FDG-PET/CT findings or the radiation dose measurements. We conducted the tumor staging according to the Union for International Cancer Control (UICC) TNM staging system for the stomach [12]. Each LN was examined employing 2 mm-spaced slices using hematoxylin-eosin-stained sections to avoid missing small focal metastases. The gastric cancers were histologically classified into two groups according to the Lauren classification system [13]. Well and moderately differentiated tubular adenocarcinoma, papillary adenocarcinoma, and solid type poorly differentiated adenocarcinoma were classified as intestinal type carcinomas. Non-solid type poorly differentiated adenocarcinoma, signet ring cell carcinoma, and mucinous carcinoma were classified as diffuse-type carcinomas.

### Statistical analysis

All statistical analyses were carried out using JMP 10.0.2 software (SAS institute, Cary, NC, USA). Differences in histological type as categorical variables were compared between metastasis-positive and metastasis-negative LNs employing the chi-square test. The Wilcoxon test was applied for continuous variables including modified SUV, the shortest LN diameter, and the time elapsed from ^18^F-FDG injection until data acquisition. Differences were considered significant at *P* < 0.05. The receiver operating characteristics (ROC) curves for the shortest LN diameter and modified SUV were used to discriminate LN metastasis from other findings. For this purpose, the area under each curve was used to measure the discriminatory ability of the model.

## Results

### Patient characteristics and PET/CT findings

In total, 21 patients were recruited for this study, and 906 LNs were harvested. Characteristics of the 21 patients are summarized in Table 1. Intestinal type was the main histopathology, being seen in 15 cases, diffuse type in the other 6. As for primary lesion, median SUV max was 7.1, and these values were higher in the intestinal type group (8.0 vs 6.4) on PET/CT scan. Median blood sugar was 95.0 mg/dL in the intestinal type group and 97.5 mg/dL in the diffuse-type group. Upon primary tumor staging of these patients, five of six (83 %) in the diffuse-type group were diagnosed as having pT4a. D2 lymphadenectomy was performed in 11 patients with intestinal type and two with diffuse-type gastric cancer. Distant LN sampling was performed in four patients because preoperative or intraoperative findings had indicated LN enlargement. Five patients with stage IV disease underwent palliative gastrectomy for anemia or symptoms of obstruction. The median primary tumor size was 6.5 cm (range, 2.3-16.5 cm). The primary lesion was larger in the diffuse-type group (11.6 vs 5.6 cm). Among 16 cases with LN metastases, 5, including 4 with intestinal type and 1 with diffuse type, had PET-positive LNs. The median SUV max of PET-positive LNs was 4.7. The sensitivity, specificity, PPV, and NPV of preoperative PET/CT were 24, 100, 100, and 57 %, respectively.

**Table 1:** Characteristics of the 21 patients and result of PET/CT

	Total	Intestinal type	Diffuse type
Characteristics	*n* = 21	*n* = 15	*n* = 6
Sex: male/female	16/5	10/5	6/0
Median age, years (range)	70 (41–81)	69 (41–81)	76 (62–81)
Operations: TG/DG/PG	11/8/2	8/6/1	4/1/1
Dissection: D0/D1/D1+/D2	1/2/5/13	1/1/2/11	0/1/3/2
Locus: upper/middle/lower	9/9/3	4/9/2	5/0/1
T status: pT1b/pT2/pT3/pT4a	3/4/6/8	2/4/6/3	1/0/0/5
N status: pN0/pN1/pN2/pN3	5/7/3/6	5/5/2/3	0/2/1/3
pStage: I/II/III/IV	2/9/5/5	1/9/2/3	1/0/3/2
SUV max of primary lesion on PET/CT			
Median (range)	7.1 (2.4-24.1)	8.0 (2.4-24.1)	6.4 (3.2-11.6)
SUV max of LNs on PET/CT			
Median (range)	4.7 (1.6-5.5)	3.15 (1.6-4.9)	5.5 (5.5)

*TG* total gastrectomy, *DG* distal gastrectomy, *PG* proximal gastrectomy, *SUV* standard uptake value, *LN* lymph node, *PET/CT* positron emission tomography/computed tomography

### Result of radiation dose measurement by well-type counter

**Fig. 1:**
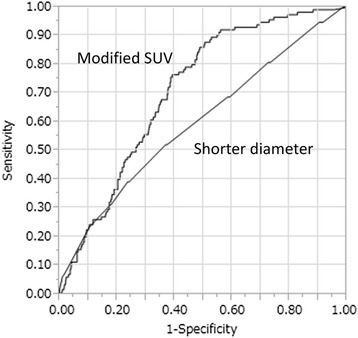
The ROC curve for modified SUV and the shortest LN diameter. The ROC curve for modified SUV had a larger area under the curve (0.71) than that for the shortest LN diameter (0.60)

**Fig. 2:**
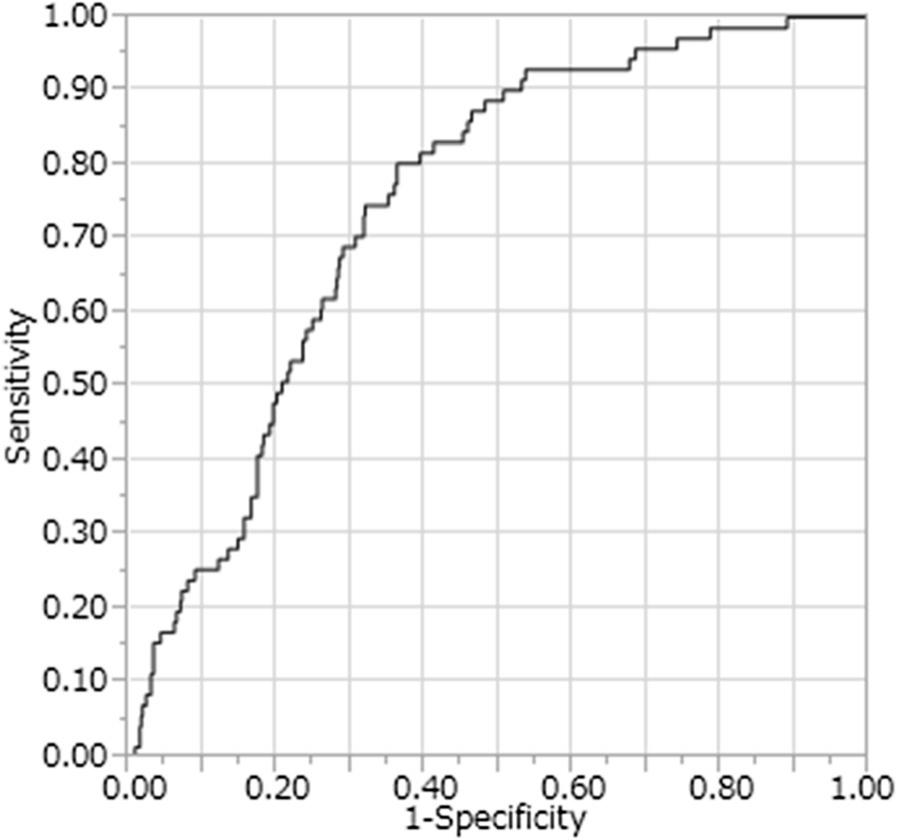
The ROC curves for modified SUV in intestinal type carcinoma. The area under the curve for this parameter is 0.75

**Fig. 3:**
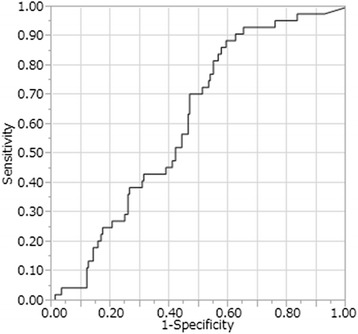
The ROC curves for modified SUV in diffuse-type carcinoma. The area under the curve for this parameter is 0.61

The 906 harvested LNs included 115 with metastases (Table 2). The median time between ^18^F-FDG injection and measurement of ^18^F-FDG uptake using the well-type counter was 444 min (range 343-527 min). Measurement by the well-type counter revealed significantly higher modified SUV among metastatic LNs than non-metastatic LNs (Table 2). The time between ^18^F-FDG injection and the completion of LN resection was 364 min in the node-positive group and 336 min in the node-negative group. The median blood sugar levels in node-positive and node-negative patients were 96 and 92 mg/dl, respectively. The time elapsed from ^18^F-FDG injection until data acquisition by well-type counter was 435 min for the metastasis-positive LNs and 446 min for the metastasis-negative LNs (*P* = 0.0003). Metastatic LNs were also significantly enlarged as compared to non-metastatic LNs (Table 2). Figure 1 shows the ROC curve for modified SUV used to distinguish between LNs with and without metastasis. The area under the curve for modified SUV was 0.71 for metastatic LNs. Using a cutoff of 2.62, the sensitivity, specificity, PPV, and NPV were 77, 60, 24, and 94 %, respectively. On the other hand, the area under the curve for the shortest LN diameter was 0.60. Using a cutoff of 7.0 mm, the sensitivity, specificity, PPV, and NPV were 39, 77, 21, and 89 %, respectively. The area under the curve was 0.75 in cases with intestinal adenocarcinoma (Fig. 2) and 0.61 in those with diffuse adenocarcinoma (Fig. 3). Using a cutoff of 2.65 in the intestinal type group, the sensitivity, specificity, PPV, and NPV were 80, 65, 23, and 96 %, respectively. In the diffuse-type group, the sensitivity, specificity, PPV, and NPV were 89, 41, 26, and 94 % with a cutoff of 1.98.

**Table 2:** Characteristics of 906 LNs in the 21 patients and measurement results by well-type counter

	LN with metastasis	LN without metastasis	*P* value
Characteristics	*n* = 115	*n* = 791	
Modified SUV			<0.001
Median (range)	3.50 (0–9.52)	2.06 (0–14.18)	
Histology of primary lesion			0.001
Intestinal	71	605	
Diffuse	44	186	
Time from ^18^F-FDG injection until data acquisition (min)			0.0003
Median (range)	435 (343–517)	446 (347–527)	
Shortest LN diameter (mm)			0.0005
Median (range)	6 (2–19)	5 (1–16)	

*LN* lymph node, ^*18*^ *F-FDG* fluorine F-18 fluorodeoxyglucose

## Discussion

To the best of our knowledge, this is the first one-to-one comparison between the ^18^F-FDG uptake and pathological status of individual LNs. Metastatic LNs showed significantly higher modified SUV than non-metastatic LNs using well-type counter. The area under the ROC curve for modified SUV was larger than that for the shortest LN diameter, indicating ^18^F-FDG uptake to be a better indicator than CT scan findings for detecting LN metastasis of gastric cancer. Micrometastasis such as isolated cluster-type tumor cells is frequently found in gastric cancer [14], therefore, accurate diagnosis of LN involvement by size is considered to be difficult. Since ^18^F-FDG uptake reflects the metabolic status of the lesion, this modality has been anticipated to be useful for the assessment of metastasis.

The area under the curve was larger for intestinal type adenocarcinoma than for the diffuse histological type. Intestinal type gastric cancer expresses more GLUT-1 than diffuse type, and this is associated with the fact that intestinal type gastric cancer shows higher SUV max than diffuse type on ^18^F-FDG PET/CT [15]. Difference of GLUT-1 expression may affect higher detection rate of ^18^F-FDG uptake for LN metastasis for intestinal type adenocarcinoma by well-type counter.

As for FDG-PET/CT, low sensitivity and high specificity of PET/CT scan for detecting LN metastasis were also demonstrated in current study. There are some reports about the sensitivity and specificity of ^18^F-FDG PET or PET/CT for detecting LN metastasis of gastric cancer [3, 6, 7, 16]. These studies included limitation of spatial resolution that ^18^F-FDG uptake in perigastric LN and inflammatory gastric wall or primary lesion could not be distinguished. Additionally, partial volume effect might affect low sensitivity of PET/CT scan. Since our one-to-one comparison was not disturbed by spatial resolution, our study revealed realistic diagnostic power of ^18^F-FDG for detecting LN metastasis of gastric cancer.

Recently, navigation surgery using a radioactive agent has been considered. Navigation surgeries are beneficial in terms of precise cancer detection, leading to avoidance of unnecessary resection. Sentinel node navigation surgery is focused on lymphatic drainage and is based on the idea that the sentinel node is the first possible site of LN metastasis [17, 18]. Navigation surgery which focuses on the metabolism of cancer cells has also been investigated [19]. Uptake of radioactive agent is detected using gamma probe without harvesting LNs; therefore, this method was expected to prevent unnecessary LN dissection. Navigation surgery using ^18^F-FDG has been reported for several malignancies [8–10]. In these series, ^18^F-FDG was injected preoperatively, and focal accumulation of ^18^F-FDG was detected with an intraoperative gamma probe. We planned this pilot study for navigation surgery using ^18^F-FDG in case with gastric cancer, but its sensitivity and specificity were not sufficient. Even though sentinel navigation surgery for early gastric cancer has 97.5 % of detection rate and 99 % of accuracy for LN evaluation, its efficacy remains to be clarified [17, 18]. Other tracers have been investigated for diagnostic application in some malignancies [20–23]; therefore, navigation surgery using these new tracers might be feasible.

This study has limitations. The first limitation was the time elapsed from ^18^F-FDG injection until radiation dose measurement. Considering the half-life of ^18^F-FDG (109.8 min), the time elapsed from ^18^F-FDG injection until data acquisition was rather long (444 min). The time elapsed from ^18^F-FDG injection until data acquisition was significantly longer for metastasis-negative than for metastasis-positive LNs. Extent of LN dissection may influence the length of this time period. Since the D2 lymphadenectomy group had more harvested LNs, it took a longer time to measure the radiation dose of all harvested LNs in this group. ^18^F-FDG uptake in tumors does not peak until approximately 4–5 h after FDG injection [19], but some of the LNs in this study showed low counts at measurement using the well-type counter. Although we normalized our data for the time factor, it may still have affected our results. We used the modified SUV to represent ^18^F-FDG uptake. This conversion facilitates correcting for various injected FDG doses and patient body masses, but some of the LNs in this study showed low counts at measurement using the well-type counter. Although we normalized our data for the time factor, our results may still have been affected. CCF was obtained by measuring activity in test tubes filled with ^18^F-FDG solution using the dose calibrator and the well-type counter, in which the radioactivity ranged from 1094 to 4 cps. The calculated CCF fluctuated more widely at the lower measured activities. Therefore, this CCF fluctuation may be one of the causes of the overlap between the positive LN and negative LN activities observed in this study. We did not investigate the correlation of SUV max on preoperative PET scans and modified SUV in postoperative analysis. Other indexes such as percent injected dose should also be investigated. The second limitation was the limited number of patients. This may affect especially low sensitivity of PET/CT scan.

## Conclusions

In conclusion, ^18^F-FDG uptake is a more useful variable than the shortest LN diameter for detecting LN metastasis of gastric cancer, especially in cases with intestinal type adenocarcinoma. However, its sensitivity and specificity were not sufficient to be applied clinically as navigation yet. Further investigation should be planned for navigation surgery.

## Abbreviations

^18^F-FDG: fluorine F-18 fluorodeoxyglucose
PET/CT: positron emission tomography/computed tomography
LN: lymph node
ROC: receiver operating characteristics
SUV: standardized uptake value
UICC: Union for International Cancer Control
ECOG-PS: European clinical oncology group performance status
FWHM: full width half-maximum
CCF: cross-calibration factor
PPV: positive predictive value
NPV: negative predictive value
